# Sexuality, fertility, family planning, family life, and partnership in young breast cancer patients: a longitudinal study

**DOI:** 10.3389/fpsyg.2023.1127359

**Published:** 2023-05-12

**Authors:** Karoline Jäkel, Diana Richter, Katja Leuteritz, Annekathrin Sender, Andreas Hinz

**Affiliations:** Department of Medical Psychology and Medical Sociology, University of Leipzig, Leipzig, Germany

**Keywords:** breast cancer, adolescent and young adult, sexuality, fertility, partnership, psycho-oncology

## Abstract

**Objectives:**

Adolescent and young adult (AYA) breast cancer patients are often faced with sexuality-related problems. Since healthcare providers are often unfamiliar with problems specific to AYA cancer this topic is too little integrated into routine oncological care. The objective of this study was to analyze sexuality, fertility, family planning, family life, and partnership regarding satisfaction and supportive care needs in AYA breast cancer patients.

**Methods:**

A total of 139 AYA breast cancer patients were examined twice, 1 year apart. The patients were asked to complete several questionnaires and to answer multiple questions about satisfaction with sexuality, fertility, family planning, family life, and corresponding supportive care needs in these domains.

**Results:**

While the patients were largely satisfied with their family life and partnerships, they were less satisfied with their sexuality and family planning. Only small mean score changes were observed in these variables over the course of a year. Being a parent already and having the possibility of further completing family planning were strongly associated with higher satisfaction and lower supportive care needs in these domains. Satisfaction was generally negatively associated with supportive care needs. Older age was predictive of lower satisfaction with sexuality at follow-up.

**Conclusion:**

AYA cancer patients deserve special consultations concerning the impact of cancer and treatment on their sexuality and fertility, and it is especially important that women who have yet to complete their family planning be actively offered information and support concerning sexuality and fertility protection before beginning treatment.

## Introduction

Breast cancer is the most frequent cancer type in women (Siegel et al., 2020; Sung et al., 2021). Due to increased screening and treatment, the 5-year survival rate of breast cancer patients has increased over the past decades [Surveillance, Epidemiology, and End Results (SEER), 2013]. However, multiple studies have shown reduced quality of life (QoL) in breast cancer patients (Engel et al., 2003; Arndt et al., 2004; Waldmann et al., 2007) and, to a lesser degree, in breast cancer survivors (Maurer et al., 2021; Roine et al., 2021). Various surgical measures, radiation therapy, or systemic therapy options (chemotherapy, antibody therapy, and anti-hormonal therapy) are available for breast cancer patients, depending on tumor stage and risk factors. These therapeutic approaches have various side effects, and it is difficult to attribute these side effects and limitations in QoL to the disease itself or to specific therapeutic measures.

While most breast cancer patients are middle-aged or elderly, interest in the issues faced by adolescent and young adult (AYA; age range: 15–39 years) cancer patients is increasing (Mütsch et al., 2019; Sender et al., 2019). Being diagnosed with cancer causes high levels of emotional distress and adverse outcomes of QoL in several social domains among this patient group (Zebrack et al., 2014; Janssen et al., 2021). In comparison with general population peers, AYA cancer patients are markedly more distressed than their older counterparts (Geue et al., 2014b; Hinz et al., 2019). This can be explained by the age-specific developmental tasks of AYAs, such as forming a partnership and family, establishing professional lives, and achieving financial independence (Zebrack and Isaacson, 2012). AYA cancer patients’ biological and clinical conditions are also different from those of older patients (Peleg Nesher et al., 2022). AYA women with breast cancer showed a higher likelihood for developing clinical depression, higher levels of distress, and lower levels of QoL when compared with older breast cancer patients (Harrison et al., 2010; Champion et al., 2014).

Many AYA cancer patients experience problems in intimate relationships (Thors et al., 2001; Fobair et al., 2006; Kedde et al., 2013; Perz et al., 2014; Reinman et al., 2021), which can lead to issues such as sexual dysfunction (Pikler and Winterowd, 2003) or loss of sexual interest associated with treatment-related body changes (Thors et al., 2001; Perz et al., 2014). In a study with young survivors of breast cancer, 55% of the women reported experiencing sexual dysfunction (Assogba et al., 2020). An Italian study with AYA breast cancer women (Biglia et al., 2010) showed that sexual activity decreased 77% after surgery, 37% 6 months after chemotherapy or endocrine therapy, and 34% 1 year later. A further longitudinal study with young breast cancer patients found that vaginal atrophy, dryness, and dyspareunia increased the risk of developing sexual dysfunction (Ganz et al., 2003). A Spanish study with breast cancer patients and a general population control group showed that the group differences in sexual satisfaction (*d* = 1.56; lower scores in the cancer group) were markedly higher than the differences in body image (*d* = 0.76) or self-esteem (*d* = 0.66; Montañés-Muro et al., 2023).

Side effects of cancer treatment can also cause fertility problems or infertility. A large population-based study in Scotland found that women were 38% less likely to conceive and carry a pregnancy to term after cancer (Anderson et al., 2018). Thus, questions of fertility and family planning are highly relevant considerations when treating young breast cancer patients (Partridge et al., 2004; Gorman et al., 2010). Although the desire to have children is a special feature of many AYA cancer patients (Schover, 2005), these patients often experience unmet needs and insufficient information concerning sexuality and fertility (Wong-Kim and Bloom, 2005; Legg et al., 2019). Studies on perceived supportive care needs (SCNs) of breast cancer patients and survivors showed that there is a specific unmet need for more information about fertility and sexuality-related issues (Thewes et al., 2004; Biglia et al., 2010). A French study found that 33% of the respondents had not received information about the impact of cancer treatment on fertility and ovarian function before starting treatment, 72% received no information about fertility preservation (Assogba et al., 2020), and only half of the AYA women with breast cancer were satisfied with the fertility- and sexuality-related information they did receive (Ben Charif et al., 2015). Even when healthcare providers recognize the importance of providing support for sexual and reproductive issues, they may face difficulties in addressing these issues (Tomioka et al., 2022).

Multiple questionnaires for measuring QoL have been developed and used with cancer patients, however, most of these do not include the domain of sexuality. Two of the most frequently used questionnaires, the Short Form Health Survey (SF-36) and the EORTC QLQ-C30, do not include this issue. This explains why knowledge about cancer patients’ sexuality and the reproductive toxicity of cancer treatment is limited when compared with other dimensions of QoL (Anderson et al., 2021).

Several studies have investigated the relationship between detriments in QoL and SCNs in cancer patients. The general result was that those patients who perceived strong detriments in a certain domain of QoL also desired more help in that domain (Colagiuri et al., 2012; Hinz et al., 2022; Lidington et al., 2022). However, the strength of this relationship was not identical for all domains. It proved to be strong in the domains of sleep problems and relatively weak in the domain of social relationships (Hinz et al., 2022). Unfortunately, these studies did not cover the domain of sexuality because it was not included in the QoL questionnaires. Therefore, the relationship between QoL and SCNs in the domain of sexuality is still to be investigated.

Satisfaction with sexuality and corresponding SCNs can change over time. A study with AYA cancer patients found that the proportion of patients who reported a negative impact of cancer on their sexual function and intimacy decreased from 48.8% to 43.4% within a one-year period (Wettergren et al., 2017), while another recent study with breast cancer patients showed a significant decrease in sexual well-being from baseline to follow-up 12 months later and a relatively stable level of sexual wellbeing in the following 12-month period (Huberts et al., 2023). These studies, however, are not focused on AYA breast cancer patients, and they only report mean score changes but not individual changes. However, even an unchanged mean score does not necessarily imply that the scores remained stable for each single patient, and the factors that determine a possible change cannot be derived. Some studies have analyzed the test–retest reliability of sexuality-related scales in cancer patients, with a time interval between the measurement points of 2 or 3 weeks (Bartula and Sherman, 2015; Mancha et al., 2019), but those analyses of test–retest reliability do not inform about medium-term changes. Moreover, changes in the mean levels of sexuality-related variables do not specify the reasons for such changes in terms of prognostic factors.

Sexuality is related to fertility, family planning, relationship with a partner, and family life, in terms of satisfaction, perceived problems, and supportive care needs. In this article, we intend to describe all these aspects and to describe their mutual relationships. In particular, the objectives of this study were (a) to analyze satisfaction with sexuality, family planning, family life, and partnership in a group of young breast cancer patients, including changes over a one-year period, (b) to examine the impact of having children, completion of family planning, and fertility preserving methods (fertiprotection) on sexuality and SCNs, (c) to analyze the associations between sexuality, fertility, family life, and partnership, and (d) to examine the impact of prognostic factors on sexuality in a longitudinal design.

## Methods

### Sample of cancer patients

The AYA-LE study is a prospective psycho-oncological study with two measuring points. The main aim of this study was to investigate psychological distress, life satisfaction, and QoL in AYA cancer patients. Patients were recruited over a period of 20 months in cooperation with 16 oncological hospitals, two local tumor registries, and four rehabilitation clinics. Study inclusion criteria were (a) age at diagnosis between 18 and 39 years, and (b) first diagnosis of a cancer at any tumor site and diagnosis within the previous 48 months. Overall, 762 patients received study information and gave written informed consent. One hundred and eighty-five of them could not be included either because they declined to participate (*n* = 43), did not meet the inclusion criteria (*n* = 88), or did not respond (*n* = 54). In all, 577 patients could be included in the study. Eleven months after the first assessment (t1), the participants were contacted again and invited to complete the questionnaires for a second time (t2). Further information on recruitment is given elsewhere (Leuteritz et al., 2018). Our analyses were restricted to female breast cancer patients (*n* = 139). The study participants responded either online or via paper questionnaires. All participants gave informed consent. The study was approved by the Ethics Committee of the University of Leipzig, reference number 372-13-16,122,013.

### Measures and variables

All sociodemographic (e.g., age, education) and clinical variables (e.g., treatment, time since diagnosis) were collected via self-report. The women were asked (yes/no-questions) as to whether they had children, a long-term partner, had completed their family planning by the time they were diagnosed, and if they had used any methods of fertiprotection. Regarding the actual wish of having a child, there were five answer options, from 1 = very weak to 5 = very strong. To assess satisfaction with different areas of life and support needs, we used established questionnaires, sometimes with minor extensions regarding sexuality and reproductive health.

### Questions of life satisfaction

The QLS questionnaire Questions of Life Satisfaction [QLS; German: Fragebogen zur Lebenszufriedenheit-Module, FLZ-M (Henrich and Herschbach, 2000)] is comprised of eight domains that represent different areas of life: friends, leisure time, health, income, work, housing, family life, and partnership/sexuality. One item example is: “How satisfied are you with your health?.” Respondents are asked to rate their level of satisfaction with these life domains on a 5-point Likert scale from 1 = very dissatisfied to 5 = very satisfied. The internal consistency of the total score of the questionnaire is Cronbach’s alpha = 0.84 (Henrich and Herschbach, 2000). In our study, we divided the originally combined domain “partnership and sexuality” into two different questions (one for partnership and one for sexuality), because the experiences with cancer patients showed that satisfaction with sexuality may be quite different from satisfaction with the partner in those samples. Moreover, we added a question regarding satisfaction with children/family planning because we were specifically interested in this issue. In our study, we used the following four scales: family life, children/family planning, partnership, and sexuality.

For some statistical analyses, the items were also dichotomized into two categories: 1–2 (dissatisfied) and 3–5 (at least moderately satisfied).

### Life satisfaction questionnaire

The Life Satisfaction Questionnaire (LSQ; German: Fragebogen zur Lebenszufriedenheit; Fahrenberg et al., 2000) was designed to assess life satisfaction in 10 dimensions. Each scale of the questionnaire consists of seven items, each of which is rated on a 7-point Likert scale ranging from 1 = very dissatisfied to 7 = very satisfied. The scale score is the sum of the item scores (range: 7–49). Higher scores represent higher levels of satisfaction. In our study, we only used the sexuality scale, which is comprised of eight the following items: sexual attraction, sexual efficiency, sexual contacts, perception of partner’s attractiveness, sexual response, sexual partner interaction, and sexual communication. One item example is “How satisfied are you with the frequency of your sexual contacts,” with the response options from 1 = very dissatisfied to 7 = very satisfied. The internal consistency of this scale is Cronbach’s alpha = 0.92 (Fahrenberg et al., 2000).

### Supportive care needs survey

The 34-item Supportive Care Needs Survey SCNS-SF34 (Boyes et al., 2009) questionnaire comprises five dimensions of perceived supportive care needs. Three of the 34 items belong to the scale of sexuality needs, one item example refers to “Changes in sexual feelings.” It is assessed whether issues of need have been experienced, which of the issues experienced remain unmet needs, and the magnitude of such needs. The answer options are: 1 = no need, not applicable; 2 = no need, satisfied; 3 = low need; 4 = moderate need, and 5 = high need.

In addition to the 34 items of the SCNS-SF34, we added one question concerning fertility and one question concerning desire for children. In this paper, we present the results of the three items of the original sexuality scale, two additional items (*fertility* and *desire for children*), and the sum scale of the three sexuality items (SCNS-Sex). This sum scale was transformed to a range of 0 to 100. For some statistical analyses, the items were also dichotomized into two categories: 1–2 (low level of SCNs) and 3–5 (at least moderate level of SCNs).

### Statistical analysis

Mean score comparisons were performed with *t*-tests, and the degree of the difference between the groups [e.g., have children (yes/no), family planning completed (yes/no), and having used methods of fertiprotection (yes/no)] was expressed in terms of effect sizes *d* according to Cohen (1988), which relate the mean score difference to the pooled standard deviation. Test–retest correlations (Pearson correlations between the t1 and t2 measurements) were calculated to test the temporal stability. Hierarchical multiple regression analyses were performed with the dependent variables SCNS-Sex and QLS at t2 and all independent sociodemographic and clinical variables (but one): *age*, *having children*, *family planning completed*, *chemotherapy*, *radiotherapy*, *hormone therapy*, and *antibody therapy*. Only fertiprotection was not included in the analyses because of the relatively high number of missing values (33%) in this variable. Two models were calculated for each of the two dependent variables: model M1 only comprised the independent sociodemographic and clinical variables mentioned above, and model M2 added the t1 score of the dependent variables to the other independent variables. The t2 scores of the dependent variables were chosen because we were interested in the possibility to predict the outcome variable in the course of 1 year. All statistics were performed with SPSS version 27.

## Results

### Characteristics of the sample

Sociodemographic and clinical characteristics of the sample of 139 breast cancer patients at t1 are presented in Table 1. The mean time since diagnosis was 11.9 months, and the mean age was 32.7 years (SD = 4.4 years, range: 20–39 years).

**Table 1:** Sample characteristics.

Variable	*n*	%
**Age group**		
<30 years	44	31.7
31–39 years	95	68.3
**Time since diagnosis**		
≤6 months	16	11.5
>6 months	123	88.5
**Education**		
≤10 years	7	5.1
11–12 years	44	31.8
>12 years	87	63.1
**Employment**		
No	23	16.5
Yes	116	83.5
**Chemotherapy**		
No	15	10.8
Yes	124	89.2
**Radiation**		
No	37	26.6
Yes	102	73.4
**Surgery**		
No	12	8.6
Yes	127	91.4
**Hormone therapy**		
No	62	44.6
Yes	77	55.4
**Antibody therapy**		
No	102	73.4
Yes	37	26.6
**Metastases** ^**(a)**^		
No	114	83.2
Yes	23	16.8
**Living with partner** ^**(a)**^		
No	29	21.3
Yes	107	78.7
**Own children**		
No	73	52.5
Yes	66	47.5
**Family planning complete** ^**(a,b)**^		
No	95	69.3
Yes	42	30.7
**Fertility preservation** ^**(a)**^		
No	54	58.1
Yes	39	41.9

^(a)^Missing data not reported; ^(b)^at time of diagnosis.

### Satisfaction with sexuality, family planning, family life, and partnership

The first research question concerned the degree of satisfaction with sexuality-related and family-related areas and the corresponding SCNs. Mean scores of the variables on sexuality and family life as well as the percentages of participants above the cutoff points are given in Table 2. According to the QLS, the patients were most satisfied with the domains *family life* (M = 3.85 at t1; item range 1–5) and *partnership* (M = 3.70 at t1), and less satisfied with *children/family planning* (M = 2.88) and *sexuality* (M = 3.03). This is also reflected in the percentages of participants above the cutoff: more than 80% were at least somewhat satisfied with *family life* and with *partnership* at t1.

**Table 2:** Mean scores, standard deviations, and temporal stability coefficients of variables concerning satisfaction with sexuality and family life.

		QLS				SCNs						LSQ
		Family life	Children Family planning	Partnership	Sexuality	Change in sex.	Change in partn.	Info about sex.	Own fertility	Desire for children	Sum	Sum
**t1**												
M (SD)		3.85 (1.11)	2.88 (1.60)	3.70 (1.36)	3.03 (1.30)	2.66 (1.67)	2.50 (1.61)	2.29 (1.44)	2.25 (1.34)	2.49 (1.66)	37.1 (35.2)	28.8 (10.0)
% above cutoff		85.3	52.3	80.1	65.4	50.4	46.8	42.4	33.8	40.3	-^(a)^	-^(a)^
**t2**												
M (SD)		3.82 (1.06)	3.06 (1.54)	3.61 (1.36)	2.93 (1.43)	2.67 (1.61)	2.40 (1.49)	2.28 (1.50)	2.19 (1.50)	2.42 (1.69)	36.3 (33.6)	29.0 (10.8)
% above cutoff		89.0	60.0	77.4	53.3	52.5	46.8	42.4	35.2	40.3	-^(a)^	-^(a)^
Difference t2 – t1		−0.03	0.18	−0.09	−0.07	0.01	−0.10	−0.01	−0.06	−0.07	−0.80	0.20
*d*		−0.03	0.11	−0.07	−0.05	0.01	−0.06	−0.01	−0.04	−0.04	−0.02	0.02
*p*		0.719	0.230	0.373	0.539	0.962	0.511	0.957	0.731	0.653	0.795	0.964
												
*r_tt_*		0.62	0.67	0.64	0.56	0.42	0.42	0.42	0.04	0.49	0.47	0.56
												
**Having children**												
No (*n* = 73)	M (SD)	3.76 (1.07)	2.12 (1.22)	3.56 (1.50)	2.90 (1.31)	2.71 (1.69)	2.41 (1.57)	2.36 (1.49)	2.04 (1.27)	2.93 (1.67)	27.3 (35.8)	29.0 (10.1)
Yes (*n* = 66)	M (SD)	3.94 (1.16)	3.69 (1.57)	3.85 (1.19)	3.17 (1.28)	2.61 (1.67)	2.59 (1.66)	2.21 (1.38)	2.48 (1.38)	2.00 (1.64)	36.7 (34.8)	28.6 (10.0)
*d*		0.16	1.13	0.22	0.20	−0.06	0.11	−0.10	0.33	−0.56	0.27	−0.04
*p*		0.343	0.001	0.228	0.224	0.710	0.513	0.557	0.051	0.001	0.922	0.790
**Family planning completed**												
No (*n* = 95)	M (SD)	3.75 (1.12)	2.09 (1.22)	3.47 (1.47)	2.75 (1.26)	2.86 (1.75)	2.62 (1.68)	2.43 (1.51)	2.20 (1.38)	3.16 (1.61)	41.0 (36.7)	28.0 (10.1)
Yes (*n* = 42)	M (SD)	4.10 (1.08)	4.62 (0.70)	4.21 (0.95)	3.67 (1.16)	2.24 (1.45)	2.21 (1.44)	2.00 (1.25)	2.36 (1.25)	1.05 (0.31)	28.8 (30.7)	31.0 (9.44)
*d*		0.32	2.64	0.61	0.76	−0.65	−0.26	−0.31	0.12	−2.20	−0.36	0.31
*p*		0.095	0.001	0.003	0.001	0.004	0.175	0.106	0.528	0.001	0.062	0.113
**Fertiprotection**												
No (*n* = 54)	M (SD)	3.75 (1.12)	2.04 (1.36)	3.38 (1.54)	2.62 (1.27)	3.06 (1.77)	2.80 (1.66)	2.33 (1.54)	2.33 (1.39)	3.39 (1.66)	44.1 (37.5)	27.9 (10.0)
Yes (*n* = 39)	M (SD)	3.92 (1.04)	2.18 (1.01)	3.64 (1.35)	2.97 (1.22)	2.54 (1.70)	2.31 (1.67)	2.41 (1.52)	1.95 (1.32)	2.85 (1.46)	35.5 (36.0)	28.9 (10.0)
*d*		0.16	0.12	0.18	0.28	−0.30	−0.29	0.05	−0.28	−0.35	−0.23	0.10
*p*		0.792	0.578	0.409	0.179	0.161	0.167	0.916	0.181	0.106	0.266	0.639

M, Mean; SD, standard deviation; d, effect size; *p*, significance level; r_tt_, test–retest correlation; ^(a)^cutoffs are only defined for the items and not for the scales.

SCNs were highest for changes in *sexuality* (M = 2.66 at t1). About 50% of the women reported SCNs concerning *changes in sexuality*, while the percentages for the other domains of SCNs were lower.

The comparison between the t2 and the t1 measurements shows that there were only minor changes in the mean scores: only one of the dimensions reported in Table 2 showed a change with an effect size of more than 0.10, and the mean scores of the summarizing scores (SCNs sum score and LSQ sexuality sum score) remained nearly unchanged (*d* ≤ 0.02).

The coefficients of temporal stability *r_tt_* (test–retest correlations) were in a moderate range from 0.42 to 0.67 with one exception: SCNs concerning respondents’ own fertility showed a low degree of temporal stability (*r_tt_* = 0.04).

### The impact of having children, family planning, and fertiprotection on sexuality-related satisfaction

Patients with children differed from those with no children in two single dimensions: they had higher levels of satisfaction with *children/family planning* (3.69 vs. 2.12, *d* = 1.13) and lower SCNs concerning *desire for children* (2.00 vs. 2.93, *d* = −0.56). Satisfaction with *sexuality* (LSQ) was nearly identical for women with and without children (*d* = −0.04).

Women who had already completed their family planning were also more satisfied with *children/family planning* (*d* = 2.64), and they perceived lower SCNs in the domain *desire for children* (*d* = −2.20) in comparison with those women who had not completed their family planning. *Fertiprotection* had no strong impact on the satisfaction scores and the SCNs: no effect size exceeded the score of 0.35.

### Correlations between satisfaction and SCNs scores

The correlations between the variables are presented in Table 3. All correlations were positive within the satisfaction variables and the SCNs variables.

**Table 3:** Correlations within the sexuality items.

	QLS				SCNs						LSQ
	Family life	Children family planning	Partnership	Sexuality	Change in sex.	Change in partn.	Info about sex.	Own fertility	Desire for children	Sum	Sum
QLS: family life	-	0.21	0.57	0.51	−0.37	−0.42	−0.34	−0.28	−0.20	−0.42	0.51
QLS: children	0.32	-	0.31	0.38	−0.12	−0.10	−0.10	0.02	−0.58	−0.12	0.20
QLS: partnership	0.54	0.34	-	0.64	−0.30	−0.32	−0.17	−0.21	−0.20	−0.30	0.54
QLS: sexuality	0.43	0.36	0.60	-	−0.57	−0.49	−0.51	−0.32	−0.30	−0.59	0.80
SCNS: changes in sexuality	−0.39	−0.26	−0.37	−0.67	-	0.78	0.70	0.52	0.40	0.93	−0.58
SCNS: changes in partnership	−0.51	−0.24	−0.54	−0.53	0.67	-	0.60	0.52	0.34	0.90	−0.56
SCNS: info about sexuality	−0.40	−0.22	−0.29	−0.55	0.75	0.53	-	0.38	0.39	0.85	−0.51
SCNS: own fertility	−0.30	−0.50	−0.21	−0.17	0.34	0.35	0.31	-	0.22	0.53	−0.47
SCNS: desire for children	−0.21	−0.70	−0.20	−0.22	0.27	0.32	0.31	0.66	-	0.42	−0.21
SCNS: sum	−0.49	−0.28	−0.46	−0.67	0.93	0.83	0.87	0.38	0.34	-	−0.62
LSQ: sexuality	0.31	0.22	0.45	0.76	−0.56	−0.46	−0.46	−0.03	−0.06	−0.57	-

Right upper triangle matrix: t1 correlations; left lower triangle matrix: t2 correlations.

High satisfaction was generally associated with lower levels of SCNs, as reflected in negative correlations in Table 3. The sexuality sum score of the LSQ was positively associated with all satisfaction domains of the QLS and negatively with all SCNs dimensions.

### Prognostic factors for sexuality variables at t2

The results of the multiple regression analyses are presented in Table 4. The dependent variables were the two scales for sexuality (SCN-Sex and LSQ-Sex) at t2. In model M1, sociodemographic and clinical variables served as independent variables, while in model M2, these variables were supplemented with the t1 score of the dependent variable.

**Table 4:** Regression of t2 sexuality scores on sociodemographic and clinical factors.

	SCN-sex				LSQ-sex			
	Model 1		Model 2		Model 1		Model 2	
	Beta	*p*	Beta	*p*	Beta	*p*	Beta	*p*
Age	−0.054	0.618	−0.069	0.480	−0.209	0.054	−0.189	0.043
Having children	0.064	0.529	0.027	0.767	−0.006	0.949	0.008	0.927
Family planning completed	−0.193	0.068	−0.095	0.318	0.155	0.136	0.078	0.387
Chemotherapy	−0.155	0.107	−0.087	0.316	0.056	0.556	0.063	0.441
Radiotherapy	0.046	0.604	0.065	0.412	−0.209	0.018	−0.169	0.026
Hormone therapy	0.053	0.568	0.047	0.572	−0.108	0.243	−0.111	0.155
Antibody therapy	0.004	0.966	0.048	0.556	−0.079	0.370	−0.025	0.736
Dependent variable at t1			0.459	<0.001			0.539	<0.001
R^2^	0.067		0.262		0.115		0.403	

Regarding model M1, the sociodemographic and clinical variables explained 6.7 and 11.5% of the variance of the dependent variables SCN-Sex and LSQ-Sex, respectively. The inclusion of the t1 scores of the dependent variables (model M2) increased the explained variance to 26.2% and 40.3%, respectively. In Model 2, none of the sociodemographic and clinical variables had a significant effect on SCN-Sex at t2, while *age* (lower LSQ scores with increasing age, beta = −0.189) and *radiotherapy* (lower LSQ score for women receiving radiotherapy, beta = −0.167) had significant effects on the prediction of the LSQ-Sex score at t2.

## Discussion

The first aim of this study was to examine satisfaction with sexuality, family planning, family life, and partnership in a group of young breast cancer patients, including changes over a one-year period. While their satisfaction with the domains *family life* and *partnership* was relatively high (M = 3.85 and M = 3.70 at t1 on a 1–5 scale, respectively), their satisfaction with *family planning* and *sexuality* was lower (M = 2.88 and M = 3.03). The relatively low level of satisfaction with sexuality in comparison with satisfaction with partnership supports the findings of a study in which 90% of the cancer patients were satisfied with their partnership but only 60% with sexual life (Lohmann et al., 2022). Particularly women without breast-conserving therapy suffer from a changed body image, which can have a negative effect on sexual feelings, and hormonal changes can also negatively affect sexual satisfaction. This is an initial indication that problems of sexuality and family planning in AYA breast cancer patients should be taken into consideration by healthcare providers.

There were only small and non-significant mean score changes over the course of the one-year period studied. Other longitudinal studies have also failed to detect significant changes over multiple years in these variables (Bradford et al., 2022), indicating that problems concerning sexuality do not disappear over time.

Concerning the temporal stability of the satisfaction and SCNs scores over time, all test–retest correlations were in the moderate range from 0.42 to 0.67 with one exception: the SCNs in the domain *desire for children* showed a correlation of only 0.04. Though the mean score of this dimension remained nearly unchanged, the low correlation means that women who had no SCNs in this dimension at t1 could potentially develop them at t2 and vice versa. Thus, it is still worth asking patients one or more years after their first consultation whether they have needs concerning their fertility and desire to have children.

As was to be expected, women who already have children were significantly more satisfied with the domains *children* and *family planning* (*d* = 1.13) than women who do not, and their SCNs concerning children were lower (*d* = −0.56). A similar and even stronger result was obtained when women who had completed their family planning were compared with women who had yet to do so. The effect sizes of the group differences were extremely high (*d* > 2.0) both for the variable *satisfaction with children and family planning* and SCNs concerning *desire for children*. This supports previous studies’ findings that it is especially important to provide information and support concerning issues of fertility to women who plan to have children (Thewes et al., 2005). A German study found out that 74% of newly diagnosed AYA patients currently wanted to have children (Geue et al., 2014a). Healthcare providers should pay particular attention to childless women hoping to have children in the future. The lack of significant differences between patients with and without fertility protection in terms of satisfaction scores may be due to the fact that, on the one hand, fertility protection means that patients are dissatisfied with their current family planning situation, but on the other hand, they hope that this can be improved by fertility protection methods.

A review on fertility and sexuality in young female cancer patients concludes that patients should be informed of potential effects of treatment before starting prescribed regimens, that fertility protection options should be explained and discussed with the patients, and that possible effects of treatments on sexual functioning should be discussed to achieve a high QoL (Condorelli et al., 2019) and to open a window for fertility protection or assisted reproductive treatment (Anazado et al., 2021). The implementation of oncofertility programs where oncologists and reproductive endocrinologists collaborate on breast cancer patients’ treatment regimens has shown that patients value fertility in the context of their cancer treatment and that they often do not voice these concerns unless prompted to do so (Vu et al., 2017).

The correlations between the satisfaction scores and the SCNs were negative: high levels of perceived SCNs are associated with low levels of satisfaction. Meeting those needs should contribute to an improvement in patients’ satisfaction. A study with AYA breast cancer patients also showed that the need to receive information on sexuality and fertility was especially high in women who had yet to complete their family planning or who had poor QoL (Thewes et al., 2005). In this context, it is interesting to note that sexual function (not satisfaction with sexuality) was unrelated to care needs related to sexuality with a correlation of *r* = 0.04 (Benedict et al., 2022).

Of the correlations between the LSQ *satisfaction with sexuality* scale and the other variables, the highest association was found for the QLS *sexuality* item (*r* = 0.80), a finding that supports the scales’ reliability.

The test–retest correlations between the t1 and t2 measurements resulted in coefficients of moderate height (between 0.42 and 0.67), with the exception of SCNs regarding their own fertility (*r* = 0.04). This means that even if no mean score change is observed in the total sample, individual changes may occur. These correlation coefficients should not be interpreted in terms of reliability since they may reflect real changes and not only a type of measurement error. Since all the test–retest correlations indicated a variance explanation (*r*^2^) of less than 50%, it is worth assessing the SCNs of AYA breast cancer patients concerning sexuality once more after a one-year period.

The regression analyses showed that *age* and *radiotherapy* are predictive of *satisfaction with sexuality* at t2, even after controlling for baseline satisfaction. The negative beta coefficient of −0.189 means that older age is associated with the development of a lower level of satisfaction with sexuality at t2, and that radiotherapy also contributes to reduced satisfaction with sexuality, while the other factors (family planning completed, chemotherapy, hormone therapy, and antibody therapy) had no statistically significant effect. Possible reasons for the effect of radiotherapy are that this kind of therapy may cause swelling, redness, and pain in the irradiated breast region, as well as increased vaginal dryness, which increases with age anyway, so that it accumulates due to radiotherapy and the women experience pain during sexual intercourse. This indicates that older women within the AYA age range and women receiving radiotherapy deserve special attention concerning questions of sexuality over the course of their illness and treatment.

To meet sexuality and fertility SCNs, healthcare providers should be more proactive in speaking about these issues, disseminating information, and recommending support resources and possible interventions (Duffy et al., 2005; Takahashi, 2014; Marsh et al., 2020). It is also helpful for AYA cancer patients to receive information about opportunities to interact with other patients who are in a similar situation. Since AYAs are familiar with social media, the use of this communication tool as a means of social support is gaining in importance (Donovan et al., 2021). Web-based psycho-educational intervention programs for AYA cancer patients on fertility-rated issues are effective tools for improving fertility knowledge (Huang et al., 2022; Micaux et al., 2022). Partners of AYA breast cancer patients should also be taken into consideration for improving communication and psychosocial support (Kauffmann et al., 2016; Shaffer et al., 2022).

Problems in the domains of sexuality, partnership, and children are not the only challenges AYA cancer patients face other issues such as maintaining or regaining work capacity are relevant as well (Brock et al., 2021; Leuteritz et al., 2021).

Issues of reproductive care are not only important for breast cancer survivors but also for survivors of other kinds of childhood cancer. An Australian clinic offered consultations for advice and management of reproductive issues for survivors of childhood cancer. The three most frequently reported symptoms or concerns reported by the survivors were related to fertility status, endocrine dysfunction, and contraception cycle (Anazado et al., 2021).

Longitudinal studies are necessary to assess the long-term effects of cancer on sexuality and fertility. A review on psychological, functional, and social outcomes in AYA cancer patients concluded that psychological and functional health outcomes improved, while negative effects on fertility and sexuality persisted over time (Bradford et al., 2022).

Some *limitations* of this study should be mentioned. Since it only included breast cancer patients, the generalizability to AYA with other kinds of cancer remains unclear. A recent study compared AYA cancer patients suffering from tumors of the reproductive organs, including breasts, with those of non-reproductive organs (Mütsch et al., 2019). The main finding of that study was that there were no significant differences between these groups concerning questions of sexuality, indicating that our study results are, at least to a degree, generalizable to other AYA cancer patient groups. There was no control group of healthy women in our study. Having normative data from samples of the general population could help better interpret issues specific to breast cancer patients. However, normative data of the specific aspects and SCNs examined in our study are not available. Most of the variables used in this study consist of only one item which limits the reliability of these instruments. Some questions of the QLS were tailored to the specific group under consideration. Concerning the cross-sectional correlations between sexuality-related satisfaction and SCNs, we cannot derive causal interpretations of the associations. In the regression analyses, we included only two of several possible dependent variables (SCN sex and LSQ sex), and fertility protection could not be included as a predictor variable because of a relatively high number of missing values. Finally, the one-year time interval studied is not sufficiently long to derive information on long-term changes.

Summing up, the results of this study underline the importance of sexuality and fertility in AYA breast cancer patients. Healthcare providers should address these issues at an early stage of treatment to facilitate communication with the AYA cancer patients and to integrate adequate psychosocial support into routine oncology.

## Data availability statement

The datasets presented in this article are not readily available because of data protection regulations concerning patient information (which assures participants that the data will not be passed on to third parties), but are available from the corresponding author upon reasonable request. Requests to access the datasets should be directed to AH, andreas.hinz@medizin.uni-leipzig.de.

## Ethics statement

The studies involving human participants were reviewed and approved by Ethics Committee of the University of Leipzig. The patients/participants provided their written informed consent to participate in this study.

## Author contributions

DR and AS designed the study. KJ, DR, and KL recruited patients. AH and KJ performed the statistical analyses. KJ wrote the first draft. KJ and AH wrote the final version. All authors contributed to the article and approved the submitted version.

## Funding

This study was funded by a grant from German Cancer Aid, grant number 110 948.

## Conflict of interest

The authors declare that the research was conducted in the absence of any commercial or financial relationships that could be construed as a potential conflict of interest.

## Publisher’s note

All claims expressed in this article are solely those of the authors and do not necessarily represent those of their affiliated organizations, or those of the publisher, the editors and the reviewers. Any product that may be evaluated in this article, or claim that may be made by its manufacturer, is not guaranteed or endorsed by the publisher.
